# The Apparent Discrepancy Between Social Inequality in Disability-Free and Quality-Adjusted Life Expectancy

**DOI:** 10.1016/j.jval.2026.01.026

**Published:** 2026-07

**Authors:** Ieva Skarda, James Lomas, Richard Cookson

**Affiliations:** Centre for Health Economics, University of York, Heslington, North Yorkshire, England, United Kingdom.

**Keywords:** EQ-5D-3L, EQ-5D-5L, health equity, healthy life expectancy, inequality aversion, quality-adjusted life-years

## Abstract

**Objectives:**

To examine how using binary rather than continuous adjustments for health-related quality of life (HRQOL) influences the estimated magnitude of social inequality in lifetime health and to explore the implications for equity weighting in distributional cost-effectiveness analysis.

**Methods:**

We used 2018 Health Survey for England and UK Office for National Statistics mortality and population data to compare health distributions in quality-adjusted life expectancy at birth (EuroQol 5-Dimension 5-Level instrument [EQ-5D-5L], 2018 value set), disability-free life expectancy (limiting long-term illness), and healthy life expectancy (self-assessed health), across neighborhood deprivation quintiles. Indirect equity weights were derived using Atkinson inequality aversion parameters, and implied mean HRQOL scores were cross-tabulated by age and deprivation to understand discrepancies.

**Results:**

Continuous adjustment yielded substantially smaller inequality gaps between the most and least deprived quintile groups than binary adjustments: quality-adjusted life expectancy gap 11.25, disability-free life expectancy gap 16.00, healthy life expectancy gap 18.53. Equity weights based on binary adjustments were substantially higher, especially at higher levels of health inequality aversion (4 times higher at Atkinson parameter 10). Social group HRQOL differences reflected both increased prevalence of morbidity and increased HRQOL burden of morbidity. However, the morbidity prevalence differential between groups was larger than the HRQOL differential.

**Conclusions:**

Disability-free and healthy life expectancy yield larger estimated magnitudes of health inequality than quality-adjusted life expectancy. This is due to their binary scaling, which implicitly assigns a HRQOL value of 0 (as bad as death) to disability or non-good health. A simple adjustment to narrow both ends of the binary scale renders the approaches more comparable.

## Highlights

• Binary adjustments for morbidity tend to produce substantially larger estimates of social inequality in expected lifetime health than continuous adjustments.
• We illustrate this apparent discrepancy and investigate its sources for England in 2018; we find that the health-related quality of life burden from disability and poor health was consistently greater during working age for the most socially deprived fifth compared with the least deprived.
• A simple harmonization adjustment to narrow both ends of the binary scale rendered the binary approach more comparable with the continuous approach.

## Introduction

Growing concern about social inequality in health is fueling growing demand for summary measures of health inequality for various purposes—monitoring change over time, cross-country comparisons, and evaluating the health inequality impacts of policy interventions using methods such as distributional cost-effectiveness analysis (DCEA).^1^, ^2^, ^3^ DCEA goes beyond standard cost-effectiveness analysis by adding information about the distribution of health gains and health opportunity costs across social groups within the general population. It can also go a step further by analyzing trade-offs between reducing health inequality and improving total health through the use of social welfare functions and equity weights. In 2025, the National Institute for Health and Care Excellence in England and Wales announced that it would start using DCEA to support decisions about the coverage and pricing of new health technologies, although only focusing on measuring health inequality impacts rather than also valuing them using equity weights.^4^

Measures based on mortality and life expectancy alone are incomplete because they do not capture the substantially worse morbidity experience of socially disadvantaged populations compared with socially advantaged populations. In response, an increasingly popular approach is to make an adjustment to life expectancy to allow for differences in morbidity.^5^ Broadly this is done in two main ways: (1) using a binary measure of disability or nongood health and (2) using a continuous measure of health-related quality of life (HRQOL). The binary approach is known as ‘disability-free life expectancy’ (DFLE) or ‘healthy life expectancy’ (HLE), whereas the continuous approach is known as ‘quality-adjusted life expectancy’ (QALE). Unfortunately, there is an apparent discrepancy between the two approaches: binary adjustment yields substantially larger estimates of the magnitude of health inequality than continuous adjustment.^6^^,^^7^ Dichotomizing health status inflates differences because nongood health is treated as 0 HRQOL, amplifying gaps. This is not a real discrepancy because DFLE/HLE and QALE are different concepts of lifetime health. However, these different concepts are easily confused, and phrases commonly used for their public communication, such as “healthy life expectancy,” “health-adjusted life expectancy,” “healthy lifespan,” “healthspan,” and “healthy years of life,” often remain ambiguous without further technical explanation. This apparent discrepancy can therefore generate confusing and conflicting headline messages about the magnitude of health inequality in a particular country, hindering cross-country and time-series comparisons. It also creates a potential problem for the use of “equity weights” for different social groups in DCEA. Equity weights of this kind adjust the value placed on health effects for different social groups, placing more importance on health benefits and health opportunity costs for more socially disadvantaged groups with worse lifetime health. Often, the equity weight for a social group depends on the estimated level of lifetime health for that social group relative to other groups. This is known as the “prioritarian” or “level-dependent” approach to setting equity weights and contrasts with the “rank-dependent” approach based purely on the ranking of the group in terms of social advantage and/or lifetime health. When using a level-dependent approach, such as the popular Atkinson index approach, a large discrepancy in estimated health levels potentially results in large differences in implied equity weights.

A continuous adjustment for morbidity is more informative than a binary adjustment, in the sense that a continuous index with many possible values (eg, based on 3125 possible health states in EQ-5D-5L) is more informative than a binary index with only two possible values. This means that the DFLE and HLE metrics based on a binary adjustment are not sensitive to many kinds of health improvement that the QALE metric is able to capture based on a continuous adjustment, including (1) increased length of life after becoming disabled or in nongood health, (2) improved HRQOL for a person who is disabled or not in good health but does not eliminate their disability or shift them across the boundary into good health, and (3) improved HRQOL for a person who is not disabled or is in good health but not perfect health.

However, binary data on disability or self-assessed health are often easier to obtain and link to mortality data than continuous data on HRQOL scores, especially but not only in low- and middle-income countries; therefore, disability-free and healthy life-expectancy approaches predominate in the monitoring literature.^8^ Hitherto, continuous approaches have been used for policy evaluation using DCEA. However, data availability constraints mean that DCEA analysts may find themselves in the position of needing to use binary adjustments. There is therefore a pressing need to understand and address this apparent discrepancy, especially for DCEA analysts in countries with limited HRQOL data availability but also for the wider community of health inequality analysts interested in using available data to make consistent comparisons of the magnitude of social inequality in health across countries and time periods.

This study makes four main contributions. First, it compares the impact of continuous versus binary adjustment on the estimated magnitude of social inequality in health in England, using the same data set and methods and thereby providing the first clean comparison of this kind. Second, it estimates how much difference this would make to equity weights in practice, based on the popular Atkinson index approach. Third, it explores the sources of the discrepancy by cross-tabulating the HRQOL scores implied by EQ-5D, disability and nongood health across age groups and social groups. Fourth, it proposes a simple adjustment for making the two approaches more comparable. Further research is needed, however, to calibrate and assess the performance of this simple approach in other contexts using other data sets and to improve our understanding of why different continuous HRQOL adjustment methods sometimes yield different estimates of health inequality.

### Data

The analysis uses data from the 2018 Health Survey for England (HSE). Conducted annually, HSE provides insights into the health of people in England and is used for planning National Health Service, tracking disease prevalence over time, and monitoring health and healthcare disparities.^9^ The HSE includes a core set of questions on general health and socioeconomic indicators, with additional modules in certain years. The sample we use has 7280 individuals and it is nationally representative. Other sample summary characteristics are presented in Appendix Table 1 in Supplemental Materials. More details about the sample design are reported elsewhere.^10^

The EQ-5D is a generic HRQOL measure with one item per dimension: mobility, self-care, usual activities, pain/discomfort, and anxiety/depression. Scores across dimensions are combined into a continuous index ranging from 1 (full health) to 0 (a state as bad as being dead), with negative values for states worse than death. Our analysis draws on the 5-L scale that was collected in the 2018 HSE, in which respondents reported no problems (1), slight (2), moderate (3), severe (4), or extreme problems (5) for each health dimension. To create HRQOL indexes we use the 2018 EQ-5D-5L value set for England.^11^ The absolute minimum value in this value set is bounded at −1, and in our HSE 2018 data, the lowest value we observe in practice is −0.285 and fewer than 1% of individuals report HRQOL below 0.

HSE also collects self-reported data on life-limiting illnesses or disabilities, including physical, mental, neurological, progressive, and learning conditions. More specifically, respondents are asked, “Do you have any physical or mental health condition or illness lasting, or expected to last, 12 months or more?” This is followed by a free-text description of the condition and a question about whether it limits their ability to carry out day-to-day activities. Based on these, we construct a binary indicator of disability-free life: coded 1 if no life-limiting illness is reported and 0 otherwise. HSE also records data on self-reported general health, which we use to construct a binary indicator of good health: coded 1 if respondents report very good or good health and 0 otherwise.

To calculate quality-adjusted, healthy, and disability-free life expectancies, we combine the specific health measurements with life tables based on UK Office for National Statistics mortality and population data for 2018. Deaths and population counts were disaggregated by age, sex, and small-area neighborhood (Lower Super Output Area), then linked to the 2019 Index of Multiple Deprivation (IMD). This enabled the estimation of mortality rates by sex, age, and deprivation level, as well as the construction of associated life tables. We define social advantage using the small area-level IMD index, which combines information on 7 dimensions of neighborhood deprivation: income, employment, education, health, crime, barriers to housing and services, and living environment. We adjust for age and sex because both factors strongly influence mortality and morbidity patterns over the life course in ways that can potentially interact with social deprivation.^12^^,^^13^ Our approach is therefore to stratify by age and sex when doing our underpinning analysis of morbidity and mortality patterns but then to aggregate upward to produce combined estimates of health-adjusted life expectancy at birth by social advantage group.

## Methods

To adjust life expectancy, we follow the method proposed by Love-Koh et al.^6^ This process involves combining data on life expectancy with data on the relevant health outcome—HRQOL, good health, and disability.

We first estimate regression coefficients to predict health outcomes by sex, socioeconomic status (SES), and age group (see the section Regression Analysis for Predicting Health Outcomes). We use small-area deprivation quintile group based on the 2015 English IMD index to measure SES. Using national mortality life tables, we then generate life-expectancy predictions by age, sex, and SES groups. The predicted health outcomes and life-expectancy estimates are combined using the Sullivan method, applied separately to each health measure^14^ (see the section Integration With Life-Expectancy Estimates). The combined results yield quality-adjusted, healthy, and disability-free life expectancies across different age, sex, and SES groups.

### Regression Analysis for Predicting Health Outcomes

In the base-case model, we predict HRQOL as a function of age group, sex, and SES status, using the HSE data for 2018. Our regression model takes the following form:

(1) $$EQ5D_{i}=\alpha +Age_{i}^{’}\times \beta_{1}+SES_{i}^{’}\times \beta_{2}+s_{i}$$

*EQ*5*D*_*i*_ is the EQ-5D-5L score for the individual, ***Age’*_*i*_** is a vector of binary indicators for each 5-year age-band (18 age-bands ranging covering ages from 16 to 20 to 90+), taking the value 1 if individual *i*’s age falls within the age bracket and 0 otherwise. ***SES’*_*i*_** is a vector of binary indicators characterizing the SES of individual *i*—more specifically, their level of deprivation based on 2015 English IMD (IMD) (1-5, 1 corresponds to most deprived, 5 to least deprived). The main column vectors of coefficients ***β*_1_** and ***β*_2_** estimate the effect of the sociodemographic characteristics on EQ-5D score. We run the models separately for male and female subsamples. This differs from the method used in Love-Koh et al^6^ because there the authors included a dummy to indicate biological sex. In our approach, we assumed the effect of age and disability differs between sexes. All of the models are run using the ordinary least squares; we further use the HSE survey weights.

Our aim is to test the sensitivity of the above approach with regard to the choice of health measure; therefore, we investigate alternative measures of health, in particular the disability-free and healthy life-expectancy measures. We perform this analysis on the 2018 HSE data using a similar regression specification as in Eq. (3).

More specifically, in the first case the outcome variable *DF*_*i*_ is set to 1 if the individual *i* does not have any long-standing limiting illness and 0 otherwise:

(2) $${DF}_{i}=\alpha +Age_{i}^{’}\times \beta_{1}+SES_{i}^{’}\times \beta_{2}+s_{i}$$

In the second case, the outcome variable GH_*i*_ is set to one if the individual *i* reports to be in good health and 0 otherwise:

(3) $${\text{GH}}_{i}=\alpha +Age_{i}^{’}\times \beta_{1}+SES_{i}^{’}\times \beta_{2}+s_{i}$$

### Integration With Life-Expectancy Estimates

These predicted health outcomes for each sex, SES, and age subgroup are then integrated with life-expectancy estimates using the Sullivan method, applied separately to each health measure. More specifically, for each sex and SES subgroup and for each age group *x*, we calculate our health-adjusted life-expectancy measure, denoted *HALE*_*x*_ (quality-adjusted life expectancy, disability-free life expectancy, or healthy life expectancy):

(4) $${HALE}_{x}=\frac{\sum_{i=x}^{\omega }L_{i}\cdot {\hat{h}}_{i}}{l_{x}}$$

in which *h*^ˆ^_*i*_ is the corresponding predicted health measure for age *i* from the regression analysis described in the section Regression Analysis for Predicting Health Outcomes (either ${E\hat{Q5}D}_{i}$ − health quality, ${\hat{DF}}_{i}$ − prevalence of disability-free or ${\hat{GH}}_{i}$ − prevalence in good health), and the rest of the variables describe information from the life tables: *L*_*i*_ are person-years lived in age interval *i*, *l*_*x*_ is number of survivors at age *x*, and ω is the final age group in the life table. For brevity, here, we omit subscripts for sex and SES but note that we repeat the calculation separately for each sex and SES subgroup.

### Simple Adjustment to Binary Measures

From an HRQOL perspective, the binary measures of health implicitly assign a HRQOL value of 1 to individuals without disability/in good health, and 0 otherwise. This typically underestimates HRQOL for people with disability or in nongood health, and typically overestimates it for people without disability or in good health.

We propose a simple adjustment to the standard binary measures to align them more closely with measures based on continuous HRQOL and reduce apparent discrepancies that might otherwise generate confusion when using binary measures in applications such as calculating equity weights in DCEA. We illustrate the simple adjustment in the main paper and describe more complex approaches to adjustment in the Appendix in Supplemental Materials. The simple adjustment is made in 2 stages.

First, if the interest is only in population mean health, assign to the 2 binary health states their respective population mean HRQOL scores, rather than 1 and 0. Second, if the interest is in health inequality, add a further decrement to the unhealthy state (ie, disability or not in good health) that allows for the further burden of disability in deprived groups. We propose a further decrement based on the difference in mean HRQOL in the unhealthy state between the most- and least-deprived groups.

For example, Eq. (2) can be adjusted to become the following:

(5) $$DFA_{i}=\alpha +Age_{i}^{’}\times \beta_{1}+SES_{i}^{’}\times \beta_{2}+s_{i}$$

in which *DFA*_*i*_ is the adjusted HRQOL of individual *i* based on their disability status. If the individual reports no disability this is set to population mean HRQOL for all individuals without disability. If the individual reports disability, this is set to population mean HRQOL for all individuals with disability minus a further decrement reflecting the difference in mean HRQOL between the most and last deprived groups for all individuals with disability.

Combining the health outcome predictions from this regression with life tables is done same way as previously described in the section Integration With Life-Expectancy Estimates, but using the new predictions from regressions with the adjusted data based on Eq. (2).

## Results

### Predicted Health Outcomes

In Appendix Table 2 in Supplemental Materials, we present the findings of the regression analyses described in the section Regression Analysis for Predicting Health Outcomes conducted separately for male and female subsamples, focusing on 3 main health measures: EQ-5D-5L, being disability free, and good health. Across all models, we observe that the influence of age on health is minimal and statistically insignificant for younger individuals, whereas older age exhibits a substantial and statistically significant negative impact on health. Notably, the coefficients are more pronounced when considering the 2 binary health measures (ie, being disability free and good health measures). Moreover, SES has an effect across all models, with a progressively stronger negative association observed with increasing levels of deprivation. For the most-deprived category, estimates range from −0.093 to −0.200 for males and from −0.057 to −0.171 for females. These findings suggest that, compared with the least-deprived group, males in the most-deprived category experience a 0.093-point reduction in EQ-5D scores and up to a 20 percentage point decrease in the likelihood of being in good health. Similarly, the probability of being in good health for the most-deprived females is 17.1 percentage points lower than that of the least deprived, accompanied by a 0.057-point decrease in EQ-5D scores.

### Quality-Adjusted Life Expectancy

In line with the regression results, the estimates for life expectancy vary across different measures of health. As shown in Table 1, when considering crude life expectancy at birth for both male and female samples combined, we observe a range from 78.33 years for the most-deprived group to 84.62 years for the least-deprived group, resulting in an inequality gap of 6.29 years.

**Table 1:** Inequality in health using different life expectancy measures.

Life expectancy measure	IMD 1 (most deprived)	IMD 2	IMD 3	IMD 4	IMD 5 (least deprived)	Inequality gap
Crude life expectancy	78.33	80.91	82.52	83.54	84.62	6.29
After health adjustment EQ-5D-5L	66.37	70.07	73.74	75.32	77.63	11.25
Disability free	53.36	58.87	62.47	65.66	69.36	16.00
In good health	51.43	59.94	63.96	67.57	69.96	18.53
Love-Koh et al^6^ estimates using EQ-5D-3L	63.2	67.7	70.0	73.2	75.1	11.9

Note. Unadjusted life expectancy and health-adjusted life expectancy measures across the 5 area-level deprivation groups in England (classified using IMD), as well as the corresponding inequality gaps between the least and deprived IMD groups.

Glossary: IMD indicates Index of Multiple Deprivation.

The gap between the most- and least-deprived groups widens when we account for differing health levels across the population. Using the EQ-5D-5L measure of HRQOL, we find that the quality-adjusted life expectancy for the most-deprived group is 66.37 years, whereas it is 77.63 years for the least-deprived group, resulting in an inequality gap of 11.25 QALEs. This gap is similar to but slightly smaller than the gap of 11.9 QALE reported in Love-Koh et al^6^ based on the EQ-5D-3L measure, as discussed later.

The observed inequality gap becomes substantially higher when using the 2 binary measures of health: DFLE and life expectancy in good health. Specifically, life expectancy without disability is 53.36 years for the most-deprived group and 69.36 years for the least-deprived group, resulting in a gap of 16.00 years. Life expectancy in good health is 51.43 years for the most-deprived group and 69.96 years for the least-deprived group, resulting in a gap of 18.53 years.

### Equity Weights Based on the Atkinson Social Welfare Function

To examine the implications for equity weighting under different health adjustments, we calculated implied equity weights for the worst-off (most-deprived) group compared with the best-off group, based on the Atkinson social welfare function. Commonly applied in DCEA, this function generates equity weights and enables sensitivity analyses across values of the health inequality aversion parameter.^2^ Higher values indicate greater concern for reducing health inequality between social groups.

**Figure 1:**
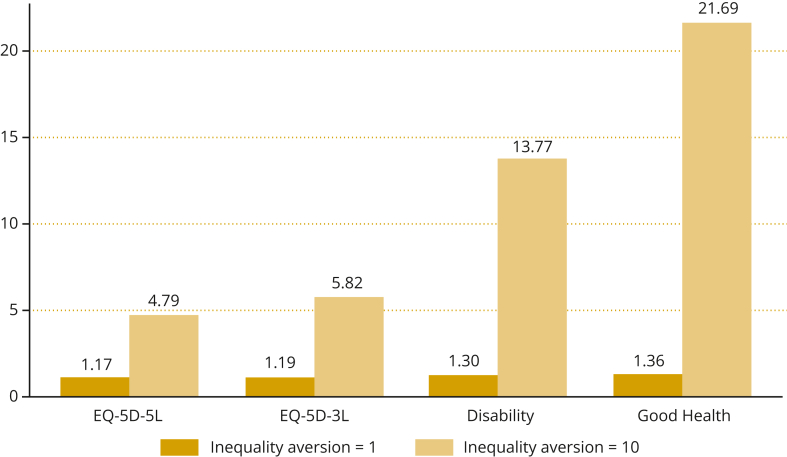
Implied equity weight for the most-deprived group across different measures of health, based on Atkinson social welfare function. *Notes:* A higher Atkinson inequality aversion parameter represents more concern for inequality: a value of 1 represents moderate concern for inequality, and a value of 10 is close to the value of 10.95, which is the estimated inequality aversion of the general public in England.^15^

Figure 1 shows implied equity weights for the worst-off group for each measure of health. When the inequality aversion parameter is set to 1, the index values are similar across all measures. However, when we increase the inequality aversion to a higher value of 10, which more closely represents the preferences of the general public in England according to a study by Robson et al,^15^ the differences become more apparent: weights range from 4.79 for EQ-5D-5L to 21.69 for good health. Thus, binary measures produce much higher weights, which in DCEA would substantially overstate the relative importance of health gains for the worst-off compared with when using the continuous measures.

### Variation in EQ-5D Scores by Age, Disability, Health, and Socioeconomic Status

**Figure 2:**
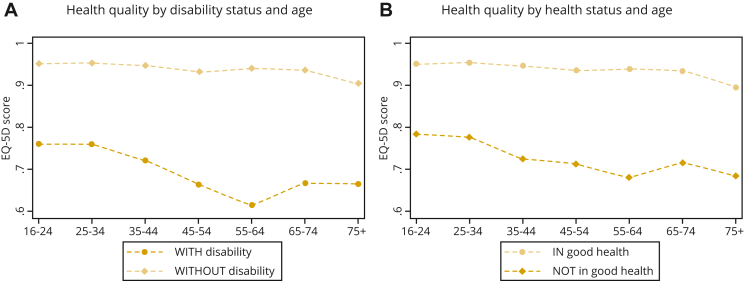
Variation in health quality by age, disability, and health status. *Notes:* EQ-5D-5L levels by age (A) for those with and without disability and (B) for those in good/not in good health.

We further investigate how EQ-5D scores vary by age, disability status, good health status, and deprivation group. Figure 2 shows the mean EQ-5D-5L score by age group for individuals with and without disabilities, as well as those who are in good health or not. The EQ-5D scores for those without disability or those who are in good health remain high (above 0.9) across all age groups. In contrast, HRQOL declines over time for both those with disabilities and those who are not in good health. The basic levels and patterns are similar across both binary health measures.

**Figure 3:**
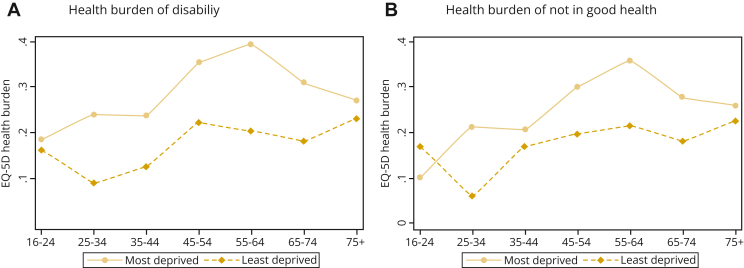
Health burden across socioeconomic status. *Notes:* (A) EQ-5D-5L health burden of having disability and (B) EQ-5D-5L burden of being in poor (not in good) health. Health burden is calculated as the mean difference in EQ-5D-5L score between (1) individuals without recorded disability and those with disability and (2) individuals in good health and those that are not in good health.

The discrepancy in the EQ-5D scores between individuals with and without disabilities, as well as between those in good health and poor health, diverges significantly between different socioeconomic strata. As evident in Figure 3, the difference is consistently greater for the most-deprived group in all age groups, except for the youngest (16-24 years). This implies that those in the most-deprived group who have disability or are not in good health encounter more significant morbidity detriments. This is presumably owing to a combination of greater severity of morbidity, greater multimorbidity (the coexistence of more than 1 chronic condition) and poorer management of chronic conditions, though we cannot disentangle causes in this study.^16^

### Simple Adjustment to the Binary Measures

According to our results, the population-weighted mean EQ-5D score for people in the “healthy” state (without disability or in good health) is about 0.95 and the corresponding score for the “unhealthy” state (with disability, or not in good health) is about 0.7, in each case (see Fig. 2). Furthermore, the disability burden is even larger for the most-deprived group by a factor of about 0.1 in each case (see Fig. 3).

Table 2 explicitly shows how the disability-free outcome from the equation (2) *DF*_*i*_ should be recoded to the adjusted outcome *DFA*_*i*_ in Eq. (5) to apply the adjustment. Our results before and after the adjustment, presented in Tables 1 and 3 show that our proposed adjustment almost completely eliminates the differences between the binary and continuous measures. When using the population-weighted (by age and deprivation) mean-reported EQ-5D score of 0.7 to estimate HRQOL for disability or nongood health, we observed a smaller inequality gap compared with the direct calculations using EQ-5D. However, when applying the additional 0.1 decrement to bring this HRQOL estimate down to 0.6 and using a simple flat adjustment of 0.6/0.95, the results are similar to the EQ-5D results.

**Table 2:** Recoding the disability-free outcome for applying the adjustment.

Disability free	${DF}_{i}$	${DFA}_{i}$
Yes	1	0.95
No	0	0.60

Note. This example illustrates how the binary disability-free outcome indicator (*DF*_*i*_) from the HSE 2018 data set was recoded to produce the adjusted disability-free outcome (*DFA*_*i*_). The adjustment aims to reduce discrepancies between binary and continuous measures of health-adjusted life expectancy.

**Table 3:** Life expectancy at birth with correction for binary measures.

	IMD 1 (most deprived)	IMD 2	IMD 3	IMD 4	IMD 5 (least deprived	Inequality gap
Adjusted disability	64.91	70.46	73.28	75.59	77.32	12.41
Adjusted in good health	65.85	69.90	72.50	74.61	77.00	11.15

Note. Health-adjusted binary life expectancy measures, after the correction described in the section Simple Adjustment to the Binary Measures. The measures are summarized across the 5 area-level deprivation groups in England (classified using IMD), followed by the corresponding inequality gaps between the least and deprived IMD groups.

Glossary: IMD indicates Index of Multiple Deprivation.

Although the simple adjustment performs well in this case, further research is needed to assess when more complex approaches may be more appropriate.

## Discussion

### Main Findings

This study compares the use of binary versus continuous measures of morbidity when producing summary measures of social inequality in lifetime health, using data from England in the year 2018.

We find that the continuous HRQOL measure, EQ-5D-5L, yields substantially smaller inequality gaps between the most- and least-deprived quintile groups compared with the binary measures of disability-free and in good health. Specifically, our estimated inequality gap in QALE using EQ-5D-5L is 11.25 years, compared with 16.00 years for DFLE and 18.53 years for HLE.

As a result, equity weights derived from binary measures are significantly higher than those based on continuous measures, particularly at higher levels of health inequality aversion. For instance, with an Atkinson inequality aversion parameter of 10—which approximates the value estimated for the general public in England—the equity weights from binary measures are approximately 4 times greater than those from continuous measures.

Social group differences in HRQOL are driven not only by increased probability of disability and poor health but also by greater severity in terms of HRQOL loss. These differences are similar for different gender and age groups, although smaller for children under 18 and for adults 75 and over. However, social group differences in the probability of disability and nongood health are larger than those in HRQOL.

We also find that a simple adjustment to disability free and HLE, based on using disability and good health to predict HRQOL, renders them more closely comparable to our quality-adjusted life-expectancy estimates based on continuous measures of HRQOL.

### Strengths and Limitations

The main strength of this study is the use of a single data source for mortality and a single data source for morbidity, with the same inequality stratification variables and estimation approach. This allows us to pinpoint the impact of health measure selection on measured inequality in a way that is independent from the data source and estimation strategy, avoiding the biases that can arise from the use of different data and methods.

The main limitation is that we have only shown that our proposed adjustment works well in this specific context, and we do not yet know whether it works well in other countries or time periods. A second issue is that the implications of this for equity weighting only matter insofar as policy makers and the public care about reducing health inequality, although there is now mounting survey evidence for this in many countries.^17^ Finally, another limitation is that the data are from before the Covid-19 pandemic, which had enduring impacts on the social patterning of health; therefore, we encourage researchers to replicate this work using more recent data. Unfortunately the HSE stopped collecting EQ-5D data after 2018, but other data sources may be available—for example, the UK Household Longitudinal Study includes SF-12 data that can be used to estimate SF-6D indexes of HRQOL, as well as standard measures of general health that can be used to define disability and good health.

### Comparison With Other Studies

Our estimated QALE gap between the most- and least-deprived fifths of England of 11.25 is smaller than the gap of 11.9 estimated by Love-Koh et al.^6^ We suspect that this is primarily owing to the change of EQ-5D index to 5 L (2018 value set) from 3 L (1995 value set) and other measurement artifacts such as the change in method of adjustment for biological sex and year-by-year fluctuations because time-series studies using more comparable data sources show a general trend toward increased health inequality between deprivation groups in England during the 2010s.^18^ The 2018 5 L index tends to yield higher HRQOL values than the 1995 3 L index, especially for people with more severe health conditions, as illustrated in Appendix Figures 2 and 3 in Supplemental Materials. using EQ-5D-3L indexes derived from HSE 2018 data on EQ-5D-5L items. That will tend to dampen the mean HRQOL differential between more and less severely ill populations, including the differential between more- and less-deprived groups. Our QALE estimates for 2018 are similar to those in a recent English study using similar HSE and mortality data pooled across 2017 and 2018,^19^ which found a QALE gap of 11.1 compared with our estimated gap of 11.25 using data for 2018 only.

Our estimated health gaps are substantially higher when using the binary health adjustments: ie, 16.00 in DFLE and 18.53 in HLE. However, after applying a simple adjustment to the binary measures to make approximate predictions of HRQOL, we obtain gaps that are much closer to the QALE estimates—12.41 based on disability adjustment and 11.45 based on self-assessed health adjustment.

### Meaning of the Study

Measures of social inequality in lifetime health based on binary morbidity data (disabled or not, good health or not) tend to yield larger estimates of magnitude compared with measures based on continuous indexes of HRQOL. This is because using a binary indicator of disability in this way implicitly assigns a HRQOL value of 1 (full health) to people without disability and 0 (as bad as death) to people with disability. This implicit estimate is larger than the actual HRQOL burden of disability and hence inflates the estimated magnitude of social inequality in lifetime health. Our view is that continuous adjustments for morbidity are generally preferable because they are more informative, especially in the context of economic evaluation in which continuous adjustment is standard practice in line with the quality-adjusted life-year concept. However, there is a case for using binary adjustments for monitoring purposes insofar as binary data remain more readily available based on survey instruments that are less frequently modified and hence can allow more routine updating and greater time-series comparability.

We propose a simple adjustment to make measures of social inequality in lifetime health based on binary morbidity data more comparable to measures based on continuous morbidity data and find that it works well in the specific context of England in 2018. This has potentially important implications for public policy. If shown to be valid more generally, this adjustment, or a modified version of it, could be useful for analysts wishing to make more reliable and accurate health inequality comparisons across counties to help policymakers address health inequalities and improve health outcomes. This adjustment could also help to reduce confusion about the reporting of health inequality statistics that is currently generated by the reporting of substantially different health inequality gap statistics for the same country in the same year. Finally, it could also be useful for researchers seeking to set up the building blocks of DCEA in their country by estimating baseline social inequality in quality-adjusted quality of life. Some countries have suitable binary measures of morbidity for constructing measures of DFLE but lack continuous measures of HRQOL. A simple adjustment could allow them to estimate QALE in a way that is more comparable with the standard quality-adjusted life-year metric used in cost-effectiveness analysis. It merely requires information on how the continuous health measure, such as EQ-5D, relates to the binary morbidity measure, and information of this kind is available in many countries and can potentially be generalized between similar countries as a first approximation.

### Research and Policy Implications

The reliability and generalizability of this simple adjustment using other data sets and in contexts outside England is not known. Therefore, before this adjustment can be routinely applied, more research is needed to calibrate and assess its performance using other data sets and in other contexts and get a better understanding of its potential biases and limitations. At present, survey instruments suitable for constructing continuous HRQOL indexes, such as EQ-5D, SF-12, and Health Utilities Index, are rarely included in the major general-purpose national data sets that are the primary tools for international comparisons of health, such as population censuses and routine household surveys. However, special-purpose EQ-5D surveys of the whole adult population have been undertaken in many countries, often including at least some indicators of social advantage.

We also suggest further health inequality research comparing different approaches to continuous HRQOL adjustment, including use of different classification levels (eg, EQ-5D-3L versus EQ-5D-5L), different value sets (eg, the England 2018 value set for EQ-5D-5L versus the UK value set due for publication in 2026, and variants thereof), and different survey instruments (eg, EQ-5D versus SF-6D). Finally, we also recommend further qualitative research to explore public understanding of different concepts of lifetime health and find ways of distinguishing more clearly between the binary HLE concept of a “healthy year” and the continuous QALE concept. In the meantime, our own suggested way of communicating HLE, DFLE, and QALE to public audiences is as follows. HLE can be described as “how many years you can expect to live in good health,” DFLE as “how many years you can expect to live without long-term illness that limits your daily life,” and QALE as “how many health-years you can expect, after each year is scored for health quality from one (full health) down to zero (an extremely severe health condition as bad as being dead).” QALE accounts for the varying degrees of severity of different illnesses and disabilities, whereas HLE and DFLE only account for ill-health in a simple yes—no way. To illustrate the difference, consider someone who lives to age 60 before becoming disabled. Although they only have 60 disability-free years, they will likely have more than 60 health-years. This is because they will gain more health-years after age 60, although they are not in full health. On the other hand, someone with 60 disability-free years might have fewer than 60 health-years. That might happen if they had minor health problems throughout life and died at 60. These problems might not count as “disability,” or “poor health,” but they still reduce health quality.

In conclusion, we have demonstrated that binary measures of health can be adjusted to align more closely with continuous measures, potentially simplifying data collection and analysis efforts. This study sets the stage for further exploration into testing and validating this approach in other populations to see how these measures can be better integrated into research and policy, with the ultimate aim of more accurately and effectively addressing health inequality.

## Article and Author Information

**Authorship Confirmation:** All authors certify that they meet the ICMJE criteria for authorship.

**Funding/Support:** This study was funded by the NIHR Health and Social Care Delivery Research program (NIHR 130258, “Unmet Need in Equitable Healthcare Resource Allocation”). The views expressed are those of the authors and not necessarily those of the National Institute for Health Research or the Department of Health and Social Care.

**Role of the Funder/Sponsor:** The funder had no role in the design and conduct of the study; collection, management, analysis, and interpretation of the data; preparation, review, or approval of the manuscript; or decision to submit the manuscript for publication.

## Author Disclosures

Author disclosure forms can be accessed below in the Supplementary Material section.

## Supplemental Material

Author Disclosures
