# Supplementary material for: The Apparent Discrepancy Between Social Inequality in Disability-Free and Quality-Adjusted Life Expectancy
Source: Value Health. 2026 Jul;29(7):1261–8. doi: 10.1016/j.jval.2026.01.026 (PMC13327847; doi:10.1016/j.jval.2026.01.026)
Supplement: Appendix [file mmc2.docx]

# Supplementary Appendix

### Additional Results and Analyses

Table A.1: Summary of key population characteristics

| ***Total sample size*** | 7,280 |
| --- | --- |
| Men | 3,513 |
| Women | 3,767 |
| ***Men by age*** |  |
| 16-24 years | 463 |
| 25-34 years | 586 |
| 35-44 years | 565 |
| 45-54 years | 639 |
| 55-64 years | 525 |
| 65-74 years | 439 |
| 75+ years | 297 |
| ***Women by age*** |  |
| 16-24 years | 451 |
| 25-34 years | 628 |
| 35-44 years | 601 |
| 45-54 years | 650 |
| 55-64 years | 578 |
| 65-74 years | 483 |
| 75+ years | 377 |
| ***By IMD group*** |  |
| Least deprived | 1,344 |
| 2nd | 1,497 |
| 3rd | 1,566 |
| 4th | 1,580 |
| Most deprived | 1,293 |

*Notes:* Data is from Health Survey for England (2018).

Table A.2: Regression results for predicting health outcomes

EQ-5D-5L Disability free In good health Estimate SE Estimate SE Estimate SE

| **Male sub-sample**  Age band (ref: 16-19) | | | | | | |
| --- | --- | --- | --- | --- | --- | --- |
| 20-24 | -0.002 | 0.015 | -0.022 | 0.041 | -0.025 | 0.04 |
| 25-29 | -0.002 | 0.014 | -0.01 | 0.04 | 0.01 | 0.036 |
| 30-34 | 0.002 | 0.013 | 0.005 | 0.036 | -0.018 | 0.035 |
| 35-39 | 0.003 | 0.013 | -0.012 | 0.036 | -0.022 | 0.035 |
| 40-44 | -0.016 | 0.015 | -0.065 | 0.039 | -0.066 | 0.037 |
| 45-49 | -0.054** | 0.017 | -0.099** | 0.038 | -0.102** | 0.037 |
| 50-54 | -0.058*** | 0.016 | -0.127*** | 0.038 | -0.169*** | 0.037 |
| 55-59 | -0.072*** | 0.017 | -0.116** | 0.038 | -0.151*** | 0.037 |
| 60-64 | -0.063*** | 0.015 | -0.166*** | 0.04 | -0.19*** | 0.039 |
| 65-69 | -0.089*** | 0.017 | -0.19*** | 0.04 | -0.212*** | 0.038 |
| 70-74 | -0.074*** | 0.015 | -0.203*** | 0.04 | -0.194*** | 0.038 |
| 75-79 | -0.111*** | 0.019 | -0.316*** | 0.045 | -0.317*** | 0.044 |
| 80-84 | -0.129*** | 0.021 | -0.415*** | 0.051 | -0.404*** | 0.05 |
| 85+ | -0.176*** | 0.024 | -0.456*** | 0.052 | -0.405*** | 0.052 |
| SES (ref: least deprived) | | | | | | |
| 2.ses | -0.016* | 0.008 | -0.04* | 0.02 | -0.026 | 0.02 |
| 3.ses | -0.04*** | 0.008 | -0.079*** | 0.021 | -0.074*** | 0.02 |
| 4.ses | -0.055*** | 0.009 | -0.102*** | 0.021 | -0.094*** | 0.02 |
| 5.ses | -0.093*** | 0.01 | -0.161*** | 0.022 | -0.2*** | 0.022 |
| Constant | 0.954*** | 0.012 | 0.908*** | 0.032 | 0.937*** | 0.031 |
| Observations | 4,046 |  | 4,509 |  | 4,509 |  |
| **Female sub-sample**  Age band (ref: 16-19) | | | | | | |
| 20-24 | -0.015 | 0.012 | 0.035 | 0.034 | -0.008 | 0.03 |
| 25-29 | -0.004 | 0.012 | -0.008 | 0.039 | -0.035 | 0.035 |
| 30-34 | 0 | 0.01 | 0.06 | 0.032 | 0.018 | 0.027 |
| 35-39 | -0.057* | 0.022 | -0.035 | 0.037 | -0.064 | 0.034 |
| 40-44 | -0.031** | 0.012 | -0.01 | 0.034 | -0.064* | 0.031 |
| 45-49 | -0.082*** | 0.016 | -0.127** | 0.04 | -0.179*** | 0.037 |
| 50-54 | -0.061*** | 0.013 | -0.087* | 0.036 | -0.176*** | 0.033 |
| 55-59 | -0.087*** | 0.015 | -0.14*** | 0.037 | -0.258*** | 0.035 |
| 60-64 | -0.098*** | 0.016 | -0.141*** | 0.037 | -0.216*** | 0.035 |
| 65-69 | -0.083*** | 0.014 | -0.223*** | 0.037 | -0.294*** | 0.034 |
| 70-74 | -0.076*** | 0.013 | -0.225*** | 0.038 | -0.296*** | 0.035 |
| 75-79 | -0.117*** | 0.017 | -0.231*** | 0.043 | -0.329*** | 0.041 |
| 80-84 | -0.141*** | 0.02 | -0.327*** | 0.052 | -0.365*** | 0.049 |
| 85+ | -0.159*** | 0.027 | -0.338*** | 0.057 | -0.464*** | 0.055 |
| SES (ref: least deprived) | | | | | | |
| 2.ses | -0.017 | 0.01 | -0.032 | 0.021 | -0.015 | 0.021 |
| 3.ses | -0.012 | 0.008 | -0.056* | 0.022 | -0.039 | 0.022 |
| 4.ses | -0.054*** | 0.01 | -0.099*** | 0.022 | -0.096*** | 0.022 |
| 5.ses | -0.057*** | 0.01 | -0.144*** | 0.023 | -0.171*** | 0.024 |
| Constant | 0.976*** | 0.009 | 0.931*** | 0.029 | 0.971*** | 0.025 |
| Observations | 3,198 |  | 3,669 |  | 3,669 |  |

*Notes:* Regression results for predicting the three health measures: EQ-5D-5L, disability free and in good health, separately for the male and female sub-samples. Data is from Health Survey for England (2018).

Figure A.1: EQ-5D and proportion of individuals in good health/without disability across socioeconomic status

## Mean EQ5D-5L score Mean EQ5D-3L score


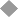

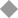

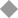

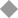

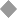

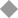

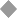

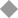

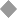


Mean score

.2

.4

.6

.8

1

Mean score

.2

.4

.6

.8

1

16-24 25-34 35-44 45-54 55-64 65-74 75+ 16-24 25-34 35-44 45-54 55-64 65-74 75+

## Proportion without disability Proportion in good health


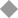

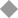

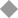

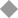

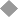

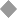

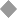

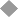

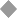

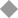

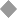


Proportion

.2

.4

.6

.8

1

Proportion

.2

.4

.6

.8

1

16-24 25-34 35-44 45-54 55-64 65-74 75+ 16-24 25-34 35-44 45-54 55-64 65-74 75+


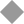

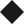


Least deprived Most deprived

Figure A.2: Scatterplot comparing EQ-5D-5L index in HSE 2018 with derived EQ-5D-3L index (based on collapsing 5L into 3L and then applying the 1995 3L value set)

*Notes:* Number of observations 7,244. Random “jitter” applied to each point, to give a clearer visual impression of the density of observations clustered at specific values, especially at the higher end.

Figure A.3: Kernel density plot comparing EQ-5D-5L index in HSE 2018 with derived EQ-5D-3L index (based on collapsing 5L into 3L and then applying the 1995 3L value set)

### Alternative Adjustment for the Binary Measure

In the main paper, we have proposed this simple adjustment, where we assume a flat adjustment of 0.6/0.95 for all socioeconomic groups. However, more complicated adjustment approaches are possible, for example, one could anchor the health of the middle socioeconomic group (with disability) to the average of 0.70 and then use a linear interpolation to model the gradient in health burden by socioeconomic group (see Table A.3). Alternatively, one could model the higher health burden of the most deprived group only (see Table A.2). In this case the simple adjustment works well, but more research is needed using data from other settings - especially low- and middle-income countries - to ascertain the circumstances in which simple versus complex approaches are appropriate and applicable given data limitations.

Table A.3: Adjustment to the binary measures: reflecting the socioeconomic gradient in health burden

| IMD deprivation group | Disability free | ${DF}_{i}$ | ${DFA}_{i}$ |
| --- | --- | --- | --- |
| 1 (most deprived) | yes | 1 | 0.950 |
| 1 (most deprived) | no | 0 | 0.650 |
| 2 | yes | 1 | 0.950 |
| 2 | no | 0 | 0.675 |
| 3 | yes | 1 | 0.950 |
| 3 | no | 0 | 0.700 |
| 4 | yes | 1 | 0.950 |
| 4 | no | 0 | 0.725 |
| 5 (least deprived) | yes | 1 | 0.950 |
| 5 (least deprived) | no | 0 | 0.750 |

*Notes:* This example illustrates an alternative approach to recode the binary indicator of disability-free outcome (*DFi* ) from the HSE 2018 dataset to produce the adjusted disability-free outcome (*DFAi* ). This approach anchors the health of the middle SES group to the population average of 0.7 and uses a linear interpolation to model the gradient in health burden.

Table A.4: Adjustment to the binary measures: heterogeneous adjustment for the most deprived group

| In the most deprived IMD group | Disability free | ${DF}_{i}$ | ${DFA}_{i}$ |
| --- | --- | --- | --- |
| no | yes | 1 | 0.95 |
| no | no | 0 | 0.70 |
| yes | yes | 1 | 0.95 |
| yes | no | 0 | 0.60 |

*Notes:* This example illustrates an alternative approach to recode the binary indicator of disability-free outcome (*DFi* ) from the HSE 2018 dataset to produce the adjusted outcome (*DFAi* ) between subgroups defined by socioeconomic status (IMD) and disability. This approach models a lower health outcome for the most deprived group in poor health.
